# Overcoming existential loneliness: a cross-cultural study

**DOI:** 10.1186/s12877-020-01753-y

**Published:** 2020-09-14

**Authors:** B. P. M. Chung, J. Olofsson, F. K. Y. Wong, M. Rämgård

**Affiliations:** 1 School of Nursing, The Hong Kong Polytechnic University, Hung Hom, Kowloon, Hong Kong, SAR China; 2 Faculty of Health and Society, Institute of Care Science, Malmo University, 25, Hus F, Malmo, Malmo, Sweden

**Keywords:** Existential loneliness, Cross-culture, Older adults, Meaning of life, Coping, Qualitative research

## Abstract

**Background:**

Moving into a long-term care facility (LTCF) can reduce the ability for older adults to engage in meaningful roles and activities and the size of their social network. These changes and losses can lead them to experience existential loneliness (EL)—the intolerable emptiness and lack of meaningful existence resulted from the losses they have experienced. While EL has often been understood as a universal human experience, it has primarily been studied in people from Western cultures; little is known about how EL may be experienced by and manifested in people from Eastern cultures. Hence, this qualitative study aimed to describe the experience and coping of EL in Hong Kong Chinese and Swedish older adults living in LTCFs.

**Methods:**

A qualitative study using Thorne’s (2004) interpretive description was conducted. Thirteen Chinese and 9 Swedes living in LTCFs in Hong Kong, China and Malmo, Sweden, respectively were interviewed about their experience of EL in two series of semi-structured interviews. Data were analyzed using thematic analysis.

**Results:**

The core theme of “overcoming EL” described the participants’ experience of EL, which came about through the combined process of “Feeling EL” and “Self-Regulating”. Both Chinese and Swedish participants had similar experience with EL. Realizing that they did not want to living with EL anymore, they coped by reframing their experience and identifying new meaning in their life.

**Conclusions:**

The study findings suggested that early and clear counselling support that help older adults to define new meaning in life may help them cope. In addition, more opportunities should be available at the LTCFs to promote quality relationships, enable older adults to reflect on their lives with pride, and support their ability to do the things they enjoy.

## Background

Many older adults reside in long-term care facilities (LTCFs) because they require assistance with their daily activities [1]. Recent data showed that among those aged 65–74 years, the demand for LTCFs ranges from less than 15% in Sweden to 50% in China [2, 3]; this demand will only increase as the world’s population ages [4]. With LTCFs being home to many older adults, it is critical that these residents experience the highest quality of life as possible [5].

LTCFs work to support their residents with their daily activities, while maintain their basic rights, freedom, choice, and dignity [6]. However, moving into a LTCF can change the way older adults see themselves and can reduce their ability to engage in meaningful experiences and roles [7]. Furthermore, their social network tends to shrink when older adults move away from their home, leading to fewer people from whom they can receive emotional support and social interactions [5, 6]. These changes and losses can lead to the feeling of loneliness [7–9].

However, this loneliness may be distinct from the more commonly studied concept of “loneliness” (i.e., having the difficulty with expressing the feeling of loneliness, the loss of social roles, or the shrinkage of one’s social network) [10–12]. Rather, older adults in LTCFs may be experiencing existential loneliness (EL)—the intolerable emptiness and lack of meaningful existence resulted from the losses they have experienced [13, 14]. EL has been correlated with serious psychological outcomes (e.g., depressive symptoms and suicidal ideation [15, 16]), and therefore, it is important to determine if older adults living in LTCFs are experiencing EL and provide support, if necessary.

Yet, there are two key limitations in the current understanding of EL. First, the idea that EL is a universal human experience was primarily based on studies of people from Western cultures [17, 18]. It is unclear if people from Eastern cultures (e.g., Chinese) also experience EL. Second, even if people from Eastern cultures also experience EL, this phenomenon may not manifest the same way as people from Eastern and Western cultures tend to experience, express, and respond to universal experiences differently or to varying degrees [19, 20]. For example, in one study that examined whether Japanese and Americans differed in self-reported emotional intensity, it was found that older Japanese people reported experiencing less intense negative emotions in response to unpleasant stimuli than their American counterparts [21]. In another study, over 400 participants from China, Japan, and the United States were assessed on their emotional intensity and self-regulation of negative emotions. This study found that the Chinese participants reported experiencing lower emotional intensity and using mood self-regulation tactics more often with than Americans [22]. These studies suggested that in order to better understand the experience of EL among older adults living in LTCFs, we need to first determine if this phenomenon is experienced the same way by people from different cultures. As such, the current study aimed to describe the experience of EL in a sample of older adults from Eastern and Western cultures who were living in LTCFs and how they cope with the experience.

## Methods

### Research questions and study design

This study aimed to answer the following questions: “How did older adults describe their experience of existential loneliness?”, “how did the older adults deal with these experiences?”, and “what were the cross-cultural similarities and differences in the EL experience?” We conducted a qualitative study using Thorne’s interpretive description [23] because it allowed the research team to examine how the study participants construct their shared experience within the context of their society and culture [24, 25].

### Participant recruitment

The research team purposively sampled Chinese (as a proxy for people from Eastern cultures) and Swedes (as a proxy for people from Western cultures) living in a government-sponsored, municipal-run LTCF in Hong Kong, China and Malmo, Sweden, respectively. These two facilities were chosen because they were the most accessible type of LTCFs for Chinese and Swedish older adults in their country due to the low fees. They also provided comparable level of care (i.e., personal and basic nursing care to dependent older adults in a communal living setting).

Residents were eligible for this study if they: (a) were aged 65 or over, (b) had been living in a LTCF for more than a year at the time of recruitment (in order to minimize confounding bias from possible negative emotional experience during the initial period of residency [26]), (c) required moderate to high level of assistance in their activities of daily living (ADL), as determined by a score of four or below in the Katz Index of Independence in Activities of Daily Living [27], (d) had been diagnosed with at least one chronic disease (e.g., heart diseases, cancer) that required regular medical follow-up and daily drug treatment, and (e) were mentally competent, based on making two errors or less on the Short Portable Mental Status Questionnaire (SPMSQ) [28]. Residents were ineligible if they: (a) were aphasic, (b) had profound hearing loss or other communication challenges, (c) were cognitively impaired (i.e., made more than two errors in the SPMSQ), and (d) were depressed (which may confound with the experience of EL), as determined by a score of two or higher on the Geriatric Depression Scale [29].

To identify and recruit participants, the researcher team, through their clinical networks, contacted the administration at the selected LTCFs to introduce the study. Once the LTCFs provided approval for the study to proceed, nurses who most often provided care at these facilities identified 20 eligible residents from each site by reviewed residents’ charts and administered the screening tools (as described above) during routine assessments. All eligible residents were then invited to an informational session where one of the authors (BC in Hong Kong and JO in Malmo) presented information about the study, answered any questions, and solicited the residents’ consent to participate.

### Data collection

Participant recruitment, data collection and analysis occurred concurrently and continued until a redundancy of patterns was achieved (‘data saturation’). Two series of in-depth, semi-structured interviews were conducted 6 months apart between March 2017 and February 2018. Participants were interviewed a second time in order to verify the initial findings based on the first set of interviews and explore any changes in their EL experience over time.

The one-on-one, in-person interviews were conducted by the authors (BC in Hong Kong and JO in Malmo) in Cantonese and Swedish, respectively. The interview guide was first pilot-tested on a Chinese and a Swedish participant, which led to only a slight rephrasing of a question to improve clarity. During the first interview, participants were asked the following questions: “Please tell me your idea about loneliness?”, “we are especially interested in a deeper sense of loneliness in life, do you have any experience on that?”, “can you recall an occasion when you felt that way?”, and “can you tell me what a day looks like when you have this feeling of existential loneliness?” Prompts were used to further explore their responses and how they handled their particular experience. At the end of each interview, the interviewer verbally summarized the participant’s responses. On average, the interviews during the first series lasted between 45 and 90 min, and around 30 min during the second series. Both series of interviews were conducted in the same conference room in each LTCF by the same interviewer. In addition, the interviewers wrote reflective notes during and within 24 h of each interview to capture the contextual characteristics, atmosphere, and relevant non-verbal expressions of the participants [30].

The interviews were audio-taped and transcribed verbatim in the original language after each interview. The transcripts were then translated word-for-word into English by the first and second author (from Chinese by BC and from Swedish by JO). When direct translation was not possible (e.g., no equivalent English words were available), the phrases were translated that aimed to capture the meaning of the EL experience and the related emotions as close to the original meaning as possible. Back-translation was done on the quotations used in the Findings section using a reflexive approach, which is a means to ensure meaning equivalence rather than seeking for word-for-word translation [31]. It shed on the complexities of ‘tentative truth claims’ constructed for EL in this study [25]. Back-translations were completed by the first author (BC) and by a bilingual Swedish colleague working in Hong Kong who has experience with qualitative nursing research but was not involved in the study. The first author (BC) and the back-translator shared the right word to use and their own perception of the text and critically reflected throughout the process [31]. All authors discussed and reached the final consensus on the translation accuracy of the quotations.

### Data analysis

Data analysis was conducted concurrently with data collection. The data was analyzed by the four-member research team that had extensive cross-disciplinary and experiential knowledge in senior care and existentialism research. Analysis began with developing the coding framework, where two members (BC and JO) independently coded three transcripts and discussed the coding with the senior researchers (FW and MR), respectively. Following the analytical methods of Thorne’s interpretive description [24], the coding process described and compared pieces of data within and across the interviews and noted similarities and differences of the ideas about the experience of EL and the ways participants coped with it. Analysis of the first three transcripts helped to establish the initial descriptive comprehension and synthesis of patterns for the coding framework. Next, critically-oriented questions were used to further probe the data; for example, the following questions were asked to the participants during the second interviews to enable further in-depth exploration of meaning [32]: “You just said sense of EL gave you time, what does it mean?” and “in what way did EL help you to reflect?”

Two research team members (BC and JO) met via videoconferencing after each transcript was analyzed and discussed the codes and patterns. During these discussions, the researchers compared commonalities and constructed categories, and then developed an interpretative account with the themes. Analytic strategies used included reflective memo-writing, re-examination of each transcript, and mapping of the codes and categories by the identified themes. A process was established to ensure at least three researchers took part in resolving analytical issues when building the coding framework. Analytic disagreements between two coders were resolved by the experienced researcher (MR) or consensus-based discussion between the four researchers until consensus was achieved.

Once the coding framework was established, all transcripts were coded using the QSR NVivo 11 data management software. The research team then conducted thematic analysis by identifying related codes and putting into themes that reflected the experience of EL in LTCFs. The team finalized the themes and subthemes into a coherent set of constructs [33], where their relationships were examined. Data analysis continued until a coherent interpretive description of participants’ experiences was achieved, as agreed by the research team. Data saturation was reached when the research team agreed that there was theoretical saturation of the analytical categories.

### Rigour

Rigour was established by adhering to the three central tenets of qualitative research: credibility, plausibility and transferability [34, 35]. Credibility was established through repeated and prolonged engagement with the interview transcripts, investigator triangulation during the development of the initial coding framework, and respondent validation of preliminary results (i.e., one Swedish and two Chinese participants were involved in confirming the findings from the first series of interviews by reviewing a summary of the interpretation with codes). This allowed participants to confirm whether the summary reflected their views, feelings, and experiences of EL. This validation allowed for clarification of and elaboration to the themes and confirmed the overall findings by the participants. Moreover, reflexivity and validation by research team members were used to ensure the credibility of the interpretations. Reflexivity was ensured through the researcher’s process of questioning (e.g., “why is this?” and “what does it mean?”). Writing reflexive memos allowed the research team to track their decision-making process when developing a more coherent analytical framework [32]. Plausibility was established through reaching data saturation, incorporating multiple perspectives (i.e., experienced clinicians and researchers in senior care), and maintaining an audit trail with memos and research journal that recorded analytic decisions. Transferability was attained through comparing the findings with existing literature.

### Ethical considerations

Ethics approval for this study in Hong Kong was obtained from the Human Subject Ethics Subcommittee of the Hong Kong Polytechnic University (HSEAR20170510001), while the approval in Sweden was provided by the Regional Ethical Review Board in Lund, Sweden (20176/781). Ethics approval was also obtained at the two LTCFs where the interviews were conducted. All participants were informed of study requirements, the voluntary nature of their participation, and their right to withdraw from the study at any time. The research team obtained informed written consent from all participants prior to study participation. Confidentiality was ensured through the use of pseudonyms in the reporting.

## Results

### Participant description

Among the 40 eligible residents identified, 13 Chinese and nine Swedes consented to participate (see Table 1 for a list of participant demographic characteristics). All consented participants took part in their first interview, and 12 Chinese and seven Swedes completed their second interview. Reasons for failure to complete the second interview included a hospital admission of a Chinese participant and the loss of contact of two Swedish participants due to their move from the participating LTCF.

**Table 1:** Demographic characteristics of study participants

Pseudonym	Sex	Current medical problems/ comorbidities	Years of staying at LTCF
**Chinese Participants**			
Angela	Female	• Stomach cancer • Diabetes (poorly controlled) • Bedridden	11
Bing	Female	• Lung cancer	9
Eddie	Male	• Lung cancer	11
Faith	Female	• Brain cancer • Hypertension (poorly controlled)	7
Grace	Female	• Stroke • Diabetes (poorly controlled)	18
John	Male	• Colon cancer	12
Kei	Female	• Ovarian cancer	8
Keung	Male	• Hyperlipidemia • Hypertension (poorly controlled) • Diabetes (poorly controlled)	5
Kwan	Male	• Hypertension (poorly controlled) • Chronic pain	15
Lee	Male	• Stroke • Bedridden	11
Mazy	Female	• End-stage breast cancer	13
Sam	Male	• Lung cancer	7
Uncle Lam	Male	• Pancreatic cancer	11
**Swedish Participants**			
Anna	Female	• Arthritis	1.5
Bella	Female	• Heart diseases	2
Bengt	Male	• Muscle degenerative diseases • Amputee (leg)	4
Bo	Male	• Heart and lung diseases	3
Britta	Female	• Scoliosis	3
Klara	Female	• Chronic lung problem	2
Nils	Male	• Stroke	14
Signe	Female	• Hypertension	3
Ture	Male	• Stroke	3

The mean age of the Chinese and Swedish participants was 82.6 and 92.4 years old, respectively. The Chinese participants had lived at their LTCF, on average, for 10.5 years, while the Swedish participants for 3.8 years. Eight Chinese and five Swedes were widowed. On average, each participant had two children. All participants (both Chinese and Swedes) lived with their family prior to their move to the LTCF; their move was a result of their increasingly poor health and need for assistance with ADLs.

### Thematic overview

**Fig. 1:**
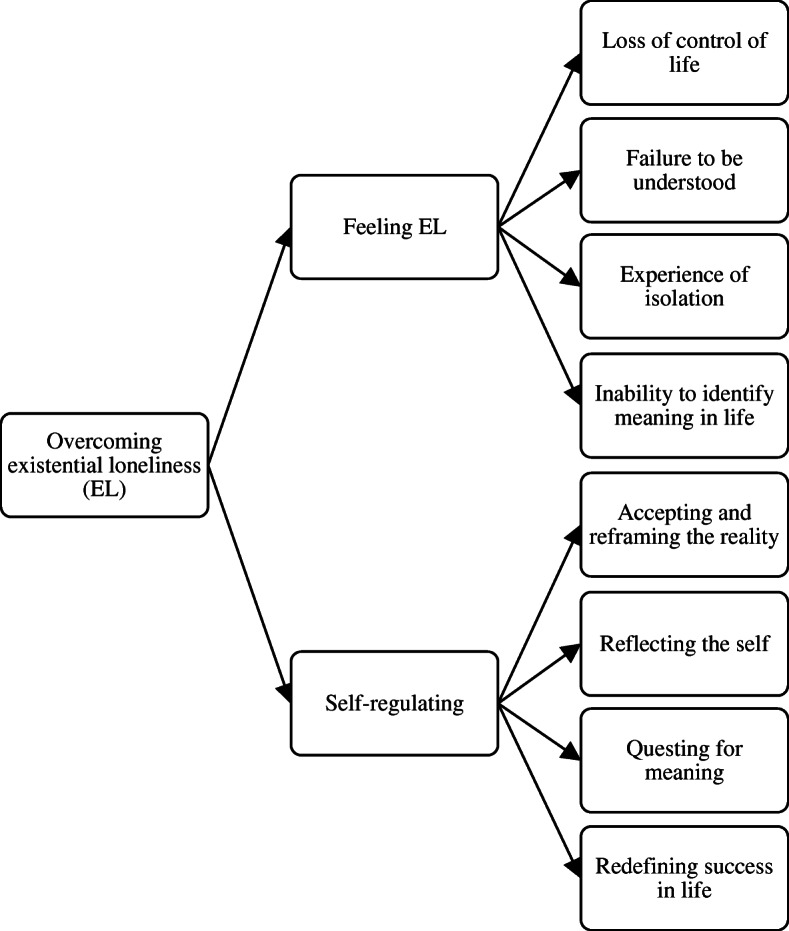
Framework of key themes and subthemes

The purpose of this study was to describe EL as experienced by Chinese and Swedish older adults living in LTCFs and the way they dealt with it. The overall theme that described both groups’ experience was “overcoming EL”, which consisted of two key themes: 1) “Feeling EL” and 2) “self-regulating” (see Fig. 1).

To overcome EL experience, the participants started by feeling the sense of loneliness and recognizing its negative impacts, then the participants consciously work to regulate their emotions. As the participants faced functional decline (e.g., frailty, illness) and their consequences (e.g., inability to care for oneself, perception of impending death), they realized the inevitability of these experiences and accepted them as part of the ageing process, similar to that of death and dying. Illnesses, frailty, and the ageing experiences represented challenges to both groups of older adults and heightened their awareness of the self. These challenges caused the participants to experience spiritual and existential distress. Their experiences were reflected in these four sequential themes: “Loss of control of life”; “failure to be understood”; “experience of isolation”; and “inability to identify meaning in life”.

Recognizing that little can be done to change the trajectory of their decline, the participants opted to cope with EL by “self-regulating” (i.e., accepting and adjusting how they feel about their situation). This act of self-regulating involved four steps: “Accepting and reframing the reality”; “reflecting the self”; “questing for meaning”; and “redefining success in life”. The process of self-regulating led the older adults to adapt to and reduce the intensity of the sense of meaningless and EL. Each of these key themes has their own sub-themes as illustrated in Fig. 1.

### Theme 1: feeling EL

To overcome EL, the participants needed to first experience it. Both Chinese and Swedish participants reported feeling worried, frustrated, and depressed as their familiar roles and life goals were disrupted. Some participants were in despair because they recognized that their life was near its end. They had accomplished their life’s tasks when they were younger. Now, they felt useless because they themselves and others assumed they could no longer contribute to life. For example, the Chinese participants felt they were no longer needed to provide and care for their children because they had grown up and able to manage their own lives. As a result, they felt they were no longer useful to their family and experienced a profound sense of loneliness, despite having family and close relatives around. They felt deserted and helpless because the experience of EL led to emotional instability, insecurity, and negativity.

When I feel lonely, I often tend to beat myself up and think that something is just wrong with me. The more alone I feel, the more I start to have thoughts of not belonging or of feeling rejected by others… The more unhappy I am, the more I avoid others and the more I isolate myself and in a lonely state… (Kei, Chinese participant)

This experience of EL was characterized by these sequential sub-themes: “Loss of control of life”; “failure to be understood”; “experience of isolation”; and “inability to identify meaning in their lives”.

#### Loss of control of life

Both Chinese and Swedish participants felt they were losing control over their own lives as they experienced physiological decline. The feeling of powerlessness took over, and they felt vulnerable.

… I cannot do what I really would like to do because of my poor health, I am forced to be sedentary for most of the day… I can’t control it. (Britta, Swedish participant)

I am old. Life is never be the same as before…with my poor health, I am unable to bathe by myself, not saying to go shopping, it is a great loss to me…I have to depend on the aids here… Being immobilized, I even can’t go to the toilet by myself and I wear napkins all the time, I have no say, no control at all… (Angela, Chinese participant)

For some participants, they felt that they had lost the right to make choices about their lives because others, like caregivers or family members, had assumed control and were making decisions for them.

Sometimes I want to take a nap but [nursing aids] say I am not allowed to...because I couldn’t fall asleep at night if I slept too much during the day! But what is there to forbid me to do things at my choice? (Bella, Swedish participant)

[The nurses] take charge of everything, they decided when and what I should eat, when I should get up and sleep, when I should void, when I should bathe, they decided everything… I lost myself… I would question myself who I am? (Angela, Chinese participant)

#### Failure to be understood

Most Chinese and Swedish participants explicitly reported feeling down, miserable, or sad because they felt like no one understood their feelings. Moreover, the participants felt that they were not being understood, despite wanting to communicate with others. This had made it impossible for them to feel connected to others.

Have you ever been in a situation when you felt like your words couldn’t get through, not being understood even talking to your own children and to your close relatives? Like you were expressing yourself over and over again, yet you were being misunderstood by your loved ones? ...I was miserable, upset, woke up at four in the morning and looking at the ceiling. (Eddie, Chinese participant)

The loneliness, I don’t want to show it my friends much as they don’t understand, I feel bad to let them know that I have the feeling of uselessness, of course, to them, this is it, this is life, they say, ‘Don’t cry, don’t cry…don’t be sad,’ and then so what? ...they don’t understand, I am still on my own, I am sad. They undermined my sense of loss. (Klara, Swedish participant)

#### Experience of isolation

Both Swedish and Chinese participants felt isolated and distanced from others and questioned where they belonged. They attributed their sense of isolation to their physical decline and the death of the beloved family members and friends.

My husband and two of my sisters do not live any longer. It is sad. They have gone...and I feel sorry for myself sometimes being the last one left in the family… My body is also starting to fall apart… I’ve got aches and pains in my shoulders, my legs, all over the body. I’m down… I’m not what I used to be. (Mazy, Chinese participant)

My children all have their own families and all managing on their own… I had two best friends when I was young but they are all gone leaving me alone. I feel I am alone… My feeling gets miserable…when my pain gets real bad and I'm hurting, and I can’t do anything hardly anymore… I am a useless person left behind… (Nils, Swedish participant)

In a few cases, Chinese participants felt depressed, despite the fact that they still had opportunities to spend time with their children.

I have to take care on my own well while I am here. If anything gone wrong…it would be the burden to my daughter. She doesn’t visit me often…she has two kids at home. She has her own job and her own family to look after…but I valued the time when she visited me with her children…sometimes I think when I have nothing that I can do for her…even make her a cup of tea, that means she doesn’t need me any longer… I am miserable. (Kei, Chinese participant)

Some Swedes felt lonely and ashamed because they had no friends.

My children always comment on my lack of friendships and do not make new friends…feel lonely that my best friends died and I have nobody to talk to. (Anna, Swedish participant).

#### Inability to identify meaning in life

Some Chinese and Swedish participants had lost their meaning in life. Their present and future were uncertain and looked bleak, and they saw how little, unworthy, and insignificant they were in their present life.

I know I’ll end up in the hospital…no future at all… Well, I don’t have to think about my children and grandchildren as I couldn’t help much in the family, I am useless now… I doubt about my ability to do anything more for them… My life is over… (Grace, Chinese participant)

I am old. Life is never be the same as before…it is different. With my poor health, it seems I have lost myself, being old is of no use…no meaning at all… (Ture, Swedish participant)

It is pointless, no meaning at all…there is nothing that I can do for myself…getting up every morning and there is nothing to do, but there are things that the others told you to do… (Nils, Swedish participant)

Under this theme of “feeling EL”, both Chinese and Swedish participants showed that EL was a common human condition and had similar characteristics across two different cultural groups. Both groups experienced EL as not being in control and understood by others, resulting in feelings of isolation and loss of meaning in life.

### Theme 2: self-regulating

The Chinese and Swedish participants coped with EL by focusing on their meaning in life and current self-identity. As the older adults recognized that their circumstance (e.g., their aging process) could not be changed and was beyond their control, they accepted that it needed to be dealt with it by developing coping strategies.

I think that something is just wrong with me. The more lonely I feel, the more I start to have thoughts of belonging to nowhere and be rejected… These thoughts further provokes my own criticism of being useless, I think worthlessness…and [there is] nothing I can do about it. I think it is my internal enemy that making me unhappy… That more unhappy I am, the more I avoid others and the more I isolate myself and in a lonely state… I but all these can’t help me out. I have to do something… (Kei, Chinese participant)

This is the fact that I am old and sick…something I can’t control…why not shifting to the things that I can do for myself now, not to think about the past nor the future… Just live in the moment. (Lee, Chinese participant)

Most of the Chinese and Swedes were struggling with their self-identity and were searching to redefine their core sense of self: “I realized my inadequacy when I am old and asked who I am?” (Bo, Swedish participant).

When the participants decided they would do something about their experience of EL, they began the process of self-regulation through the following steps: “Accepting and reframing the reality”; “reflecting the self”; “questing for meaning”; and “redefining success in life”. This process led the participants to unfold their life experience and re-examine themselves, where they looked from within and saw who they really were, thus enabling them to develop a sense of well-being.

#### Accepting and reframing the reality

Most of the Chinese and Swedish participants coped with their experience of EL by accepting their own limitations, weaknesses, and loneliness, and recognizing that change was needed. They began paying attention to the present moment rather than being trapped in the past and worrying about the future. They accepted EL in a new way by recognizing that it was unavoidable, appreciating its positive aspects, and reframing it as an opportunity. They were then able to experience life more fully and become more whole human beings: “I have to endure and live with what cannot be changed.” (Signe, Swedish participant)

I am old… Life will never be the same as before, this is it, I have to accept this is the “present me”, it always be different from the “past me”… It seems that I have lost my world as a rubbish when depending on others…but when my grandkids called me grand-pa… I am excited, I am still their grandpa, I didn’t lose my identity as a grand-pa… I should enjoy every moment with my grand-kids. This is me I enjoyed it. (Lee, Chinese participant)

The loneliness is sometimes hard to cope especially in the evenings, I know I would end up at the hospital…Well, I am by myself here. It can be positive of being myself here. I am alone to think through my own life…It is very good to get these ‘quiet’ times and think through who I am. (Britta, Swedish participant)

#### Reflecting the self

Being alone had been reframed as an opportunity to not only reflect about the past and the future, but also examine their own thoughts and feelings and what they mean to them.

I think being alone might be a chance for me to reflect, to think about the past and redefine my life purpose, not just sit there and wait for nothing… I went to the Hong Kong Museum of Coastal Defense to recapture my old days. I am proud to tell my grandson that I was a soldier in the Chinese Civil War in the 1940s. I am satisfied with my past accomplishment and my grandson said, my grandpa fight in war. (Uncle Lam, Chinese participant)

It can be a sense of positive loneliness. The positive thing is that I can be alone and think through life. It is very good to get these times and think through about who I am. (Britta, Swedish participant)

I think this might be a chance for me to reflect, think and plan my remaining days...in which I have nothing more to lose. (Keung, Chinese participant)

#### Questing for meaning

Several Chinese participants found ways to feel satisfied and regain meaning to their lives. They recognized that EL was unquestionably challenging, and it led them to some personal reflection. The participants learned to accept their EL and their feelings toward it. In doing so, the participants regulated their negative emotion by reframing their experience and identifying new opportunities.

I found it is no use to linger on the anger or get depressed, sad to being old and lonely. To me, to make my daughter worrying about me is a sin… I should focus my days on the things that I could do…it is more meaningful to me to live in a moment. (Kei, Chinese participant)

I let myself be alone but not lonely. Letting myself be this way doesn’t mean hiding from pain, loss or misery. It just means I go with the present experience and make meaning for my existence. When the sad things came, I opened up to them and let them in—to face it. I would choose to get out of here and it is my new way of control (Sam, Chinese participant)

The participants used different ways to create new meanings in their life. The Chinese participants explored their self-worth by acknowledging what they could still offer to their family and community; the Swedes focused their search for meaning through their hopes of re-enacting a new self and socializing with friends.

I just think of it as me being what I am, and what I’ve been through, and all the other stuff. It's just the loss of what you use to have, to what you don't have today, is why I am the way I am now. That’s how I look at it, I guess. (Ture, Swedish participant)

I work in the support groups here. I hope to help the others go through the experience, sadness, happiness, sorrow, hoping what I have used to support myself in those days might help them too, we have to accept we are old and have to be by ourselves. It is meaningful that I am not useless…still I can contribute…despite I don’t know how big the contribution is…it doesn’t matter. (Bing, Chinese participant)

I wake up every morning and thinking I am glad that I can get up and make myself a cup of coffee. It is good enough for myself now. (Signe, Swedish participant)

I do not feel lonely because I have friends around me here when having dinner for example and we have activities as well and it is nice and worth to spend time with friends, their presence was my support. I found my own meaning of life is to find happiness with friends (Anna, Swedish participant)

Being able to find new meaning in their current life not only helped them to identify their new reason for living, but also gave them back control of their own lives. This, in turn, reduced their sense of EL.

#### Redefining success in life

Finally, the participants saw that it was time to redefine success in their own terms. For many participants, their ‘life-is-good’ perspective was embedded in memories of past success and the present challenges.

I come to the realization that unless there is something positive, um, “life-is-not-so-bad”, that I have to just go on with what I have. There are still times that I have down days of course. That’s life. (Eddie, Chinese participant)

In particular, the Chinese participants not only defined success in terms of their own making, but also found comfort in the success of their family and community.

I know I’ll end up in the hospital…no future at all… Well, I don’t have to think about my kids and grandchildren in fact they are doing well on their own. I established the family by my own pairs, not very well-off though, it is my success, I define my success is not only to my own but to my whole family. I don’t take the negative so seriously. What is far more important is love and my family… I can’t afford to lose more… (Kwan, Chinese participant)

I spent considerable time in the groups such as Christian group, not always depended on others. I think, “something good has to come from this experience.” Actually, I didn’t know what good it would be, but the idea that I could share my experience, so that others who joined this group do not have to go through the same thing as I did in the past, this experience gave me considerable comfort, it make senses of my worth. In a way, it is my success. (Grace, Chinese participant)

For the Swedish participants, many defined their success from their own lens.

I know that my time is short…even if I had a stroke and am alone here… No, I don’t see the experience as a bad thing because, it, what I had in the past was nothing. I was nothing before then, you know. Then, why bother! I want to make clear that my acceptance does not mean giving up or being passive. It is my success to think good about myself. (Ture, Swedish participant)

## Discussion

This study described the experience of EL in Chinese and Swedish older adults living in LTCFs and highlighted the nuanced cultural similarities and differences on how Chinese and the Swedes dealt with EL. In general, both Chinese and Swedish older adults in this study described their EL experience as “overcoming” rather than “being trapped”. This is different from existing literature, where older adults from Western cultures reported their experience of EL as being “trapped in an anxious state” without meaningful interpersonal relationships, and “imprisoned” in feelings of being useless and unconnected [36]. The participants experienced EL in terms of the loss of control of life, failure to be understood, experience of isolation, and inability to identify meaning in their lives. This experience of EL appears to be similar to the concept of disrupted self—a self-based notion of aging that having a meaningful and productive life, typically through meaningful roles and life goals, contribution to family and society and being in control of their lives and their world [37].

This study set out to determine if EL is a common human experience, where both Chinese and Swedish participants experience the phenomenon in a similar way. We found that both groups characterized their EL experience as recognizing their mortality, experiencing disruptions to familiar roles and life goals, feeling useless because others (or even themselves) assumed they could no longer contribute to society, and losing control in managing their own lives. The sense of EL stemmed from the gap between the sense of meaninglessness that the participants felt about their current life and the full and productive life they used to lead. Similar experiences were reported in other populations such as the frail elderly, advanced cancer patients, dying persons and terminally ill patients [38–40]. Specifically, during the time of dramatic upheaval and instabilities where their physical and existential boundaries were challenged, participants of these studies also experienced the loss of control, feeling of being worthless, or seeking their purpose in life. However, our participants’ EL experience also included the loss of their identity and ability to perform meaningful acts. Thus, our findings suggested that the sense of emptiness and lack of meaningful existence from the aging-related losses experienced by older adults were compounded the confounding impact of the disrupted self.

Our findings were consistent with findings from other studies of dying and cancers patients from Western cultures, which showed that the suffering of EL was accompanied by a disrupted sense of identity [41, 42]. Moreover, changes in self-identity was well-articulated in our Chinese participants, where these changes were due to shifting in role and relationships with their family. The possibility of a “disrupted self” made the older adults realized that something was at stake and prompted them to reflect [36]. This is important because EL allowed our participants the time for self-reflection, which is often difficult when people are bogged down by everyday life and struggles.

Over time, the participants in this study accepted that EL was an unavoidable experience that was beyond their control, similar to the acceptance of the impact of their frailty, illness, and the ageing experiences. Recognizing that they could not continue to live with EL, the study participants chose to cope with it. Because there were no alternative ways to manage the disrupted self, these older adults turned inwards by self-regulation. Specifically, they hoped to lessen the sense of negativity and the impact of EL by accepting and reframing their experience, examining oneself, questing for meaning, and redefining success in life. Questions remained on what this self-regulation looked like as older adults moved forward to a more positive and optimistic view of their life.

These findings are consistent with studies of patients coping with terminal chronic illnesses, like cancer, neurodegenerative disorders, and multiple morbidities, who also coped by self-regulation [43–46]. Self-regulation did not represent passivity or “giving up” in both ethnic groups in our study. It required that these older adults openly recognized and confronted their limitations and the negative feelings related to EL. In addition, our participants were active agents who quested for meaning and redefined success in life, which were predictors of coping well among older adults [47, 48]. It meant that the participants needed to learn to transform the negative emotions and outcomes of EL into something that was positive and meaningful. In doing so, older adults could make sense and meaning of their EL and improve their overall well-being.

This study also explored the role of culture in relation to the experience of EL. While the findings showed that there were similarities between Chinese and Swedes in their overall EL experience, we found that the Chinese participants defined oneself through their family and their role within it, whereas the Swedes defined it were through participating in friendship and leisure activities, achieving personal accomplishment, and meeting their individual needs. This is consistent with existing literature that describes the influence of culture on self-identity [48]. For instance, the individualistic values that are typical of people from the Western cultures characterize the personal self in terms of self-enhancement and personal growth [49], while those of the Eastern cultures tend to hold collectivistic values that focus on social and family roles, obligations toward children and grandchildren, and moral behavior [50]. Future analyses should focus on how these different values imbued with ethnic undertones inform the redefining of the older self.

## Limitations

This is the first qualitative study we know of that described and compared how older adults from the Swedish and Chinese make sense of their EL experience. Some limitations should be noted. While there was some diversity in participants’ experience, negative cases where older adults did not fare well with EL were limited. Hence, the experience of “overcoming EL” may not be transferable to those whose new sources of meaning of life could not be identified. This study was conducted in the LTCFs, the findings may not be applicable to those older adults who live in the community.

## Conclusions

Our study findings shed some light on the cross-cultural experience of EL. While the overall experience of EL are comparable between the Chinese and Swedish participants, strategies that address the disrupted self will need to be tailored to how people define themselves (e.g., based on social and cultural roles vs. individual pursuits, achievements, and relationships), which in part is shaped by their cultural background. Learning from these findings, clinicians may wish to support older adults living in LTCFs to reengage in other meaningful goals and activities and facilitate identifying their new self. In addition, opportunities should be created at the LTCFs to promote quality relationships, enable older adults to look back on their lives with pride, and support their ability to do the things they enjoy.

## Data Availability

The datasets used and/or analyzed during the current study are available from the corresponding author upon request. All data and material will be made available.

## Abbreviations

ADL: Activities of Daily Living
EL: Existential Loneliness
LTCF: Long- Term Care Facilities
SPMSQ: Short Portable Mental Status Questionnaire
